# SET7/9 promotes multiple malignant processes in breast cancer development via RUNX2 activation and is negatively regulated by TRIM21

**DOI:** 10.1038/s41419-020-2350-2

**Published:** 2020-02-26

**Authors:** Wenzhe Si, Jiansuo Zhou, Yang Zhao, Jiajia Zheng, Liyan Cui

**Affiliations:** Department of Laboratory Medicine, Peking University Third Hospital, Peking University Health Science Center, Beijing, 100191 China

**Keywords:** Breast cancer, Transcription

## Abstract

Although the deregulation of lysine methyltransferase (su(var)-3–9, enhancer-of-zeste, trithorax) domain-containing protein 7/9 (SET7/9) has been identified in a variety of cancers, the potential role of SET7/9 and the molecular events in which it is involved in breast cancer remain obscure. Using the online Human Protein Atlas and GEO databases, the expression of SET7/9 was analyzed. Furthermore, we investigated the underlying mechanisms using chromatin immunoprecipitation-based deep sequencing (ChIP-seq) and quantitative ChIP assays. To explore the physiological role of SET7/9, functional analyses such as CCK-8, colony formation, and transwell assays were performed and a xenograft tumor model was generated with the human breast cancer cell lines MCF-7 and MDA-MB-231. Mass spectrometry, co-immunoprecipitation, GST pull-down, and ubiquitination assays were used to explore the mechanisms of SET7/9 function in breast cancer. We evaluated the expression of SET7/9 in different breast cancer cohorts and found that higher expression indicated worse survival times in these public databases. We demonstrated positive effects of SET7/9 on cell proliferation, migration, and invasion via the activation of Runt-related transcription factor 2 (RUNX2). We demonstrate that tripartite motif-containing protein 21 (TRIM21) physically associates with SET7/9 and functions as a major negative regulator upstream of SET7/9 through a proteasome-dependent mechanism and increased ubiquitination. Taken together, our data suggest that SET7/9 has a promoting role via the regulation of RUNX2, whereas TRIM21-mediated SET7/9 degradation acts as an anti-braking system in the progression of breast cancer.

## Introduction

Breast cancer is one of the most common cancers among females worldwide; although numerous advances in surgical resection, radiotherapy, chemotherapy, and neoadjuvant therapies have been made in recent decades, the morbidity and mortality of breast cancer remain high, and metastasis is still the major cause of cancer-related death^1–3^. New therapies based on the individual characteristics of clinical pathology have recently attracted increasing interest, so more intensive studies are needed to understand the mechanisms of breast cancer tumor progression.

SET7/9 is the first lysine methyltransferase discovered that can generate monomethylation at histone 3 lysine 4, which is a marker of transcriptional activation^4,5^. SET7/9 both has a role in regulating euchromatic gene expression through H3K4 methylation^6–8^ and can methylate a number of non-histone proteins such as UHRF1^9^, Rpl29^10^, Gli3^11^, SOX2^12^, LIN28A^13^, and FOXO^14^. How the functions of SET7/9 in transcriptional activation are coordinated and whether SET7/9 is involved in breast cancer currently remain controversial. This study aims to reveal the effect and underlying mechanism of SET7/9 in breast cancer cell proliferation, migration, and invasion using in vitro and in vivo experiments. We demonstrate that SET7/9 is a potential novel candidate therapeutic target for breast cancer therapy.

RUNX2, which is best known as a transcription factor involved in bone development^15^, is also detected in the embryonic mammary placodes of the developing mammary gland^16,17^. Like many other transcription factors, the upregulation of RUNX2 is regarded as an important contributor in breast cancer; however, the exact mechanism of RUNX2 is only beginning to be elucidated^18,19^.

Tripartite motif-containing protein 21 (TRIM21), a member of the family of tripartite motif (TRIM)-containing proteins, is a RING finger domain-containing E3 ubiquitin ligase that was first implicated in autoimmune diseases^20^. The dysregulation of TRIM21 contributes to the progression of human malignancies including breast cancer^21,22^. Our data provide compelling evidence that the degradation of SET7/9 by TRIM21 can be used to suppress breast cancer progression.

## Results

### SET7/9 is upregulated in breast cancer tissues and predicts a worse prognosis

**Fig. 1:**
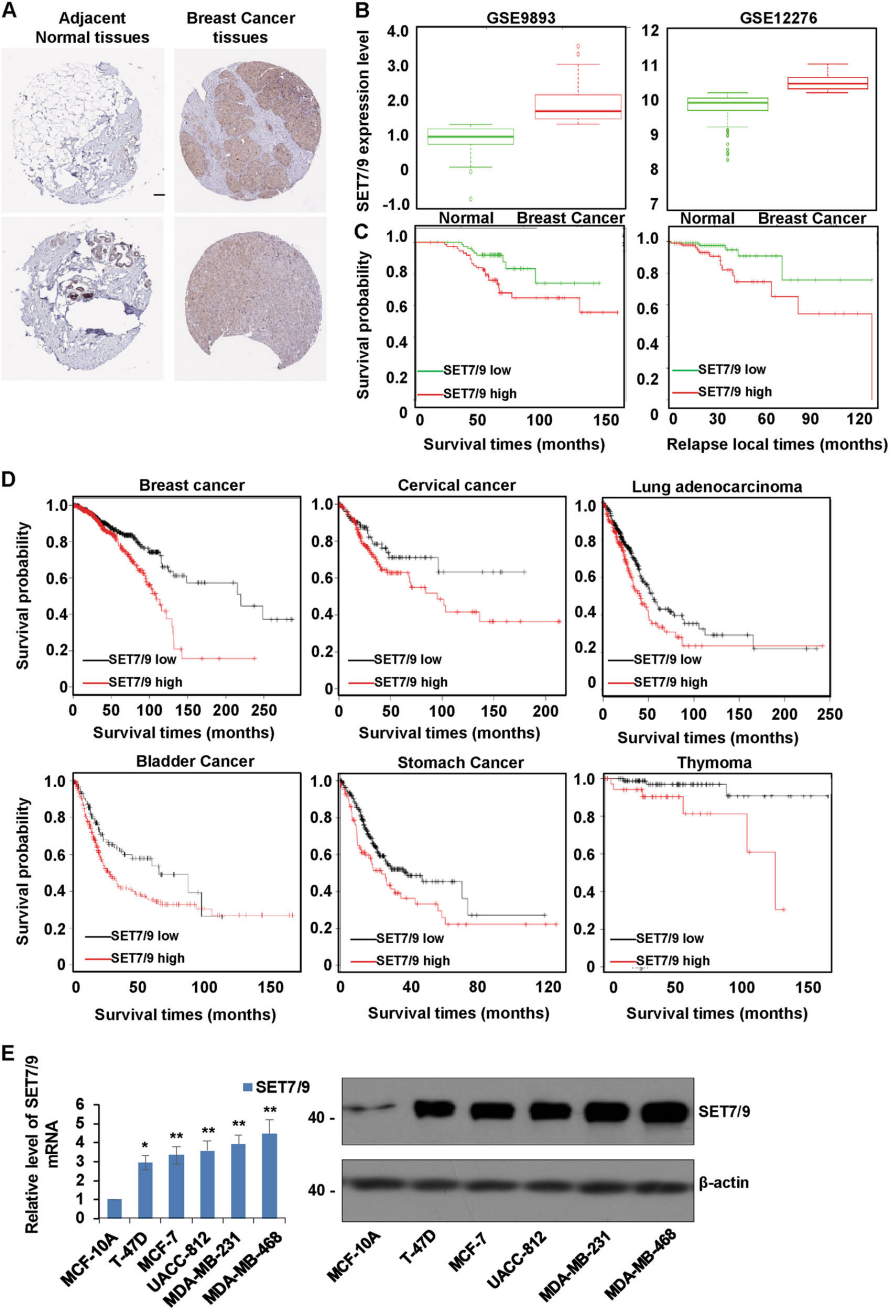
SET7/9 is upregulated in breast cancer tissues and predicts a worse prognosis. **a** The expression of SET7/9 was determined in breast cancer specimens and normal breast tissues. The images were taken from the online database of the Human Protein Atlas. **b** The relative expression of SET7/9 was measured in different breast cancer cohorts, including GSE9893 and GSE12276. **c** The overall survival times and local relapse times in the large cohort of breast cancer patients were analyzed by Kaplan–Meier analysis using the GSE9893 and GSE12276 data sets, and the log-rank *p* value is shown. **d** Kaplan–Meier plots show the association between SET7/9 mRNA expression and patient overall survival times in patients with breast cancer, cervical cancer, lung adenocarcinoma, bladder cancer, stomach cancer, and thymoma. **e** RT-qPCR and western blot analysis were used to measure the expression of SET7/9 in normal breast epithelial cells and breast cancer cells including T-47D, MCF-7, UACC-812, MDA-MB-231, and MDA-MB-468. Values are mean ± S.D. *n* = 3. *P < 0.05, **P < 0.01.

Using immunohistochemical staining data from the online database of the Human Protein Atlas, we found that SET7/9 displayed strong positive expression in breast cancer tissues and weak expression in normal breast tissues (Fig. 1a). Accordingly, by analyzing the Gene Expression Omnibus (GEO) GSE9893 and GSE12276 databases, the expression of SET7/9 was explored in different cohorts, and we noted that SET7/9 expression was significantly higher in breast tumor tissues and had negative effects on patient overall survival rates and local relapse times (Fig. 1b, c). Similarly, through online survival analysis with the Kaplan–Meier plotter, the prognostic value of SET7/9 mRNA in breast cancer was demonstrated according to RNA-seq data. Elevated SET7/9 mRNA expression was associated with shorter overall survival times (hazard ratio, 1.81; *p* = 0.00031). Moreover, increased expression levels of SET7/9 were related to adverse prognostic values in cervical cancer, lung adenocarcinoma, bladder cancer, stomach cancer, and thymoma (Fig. 1d). Likewise, the mRNA and protein expression of SET7/9 was higher in breast cancer cells than in MCF-10A normal mammary gland epithelial cells (Fig. 1e).

### Identification of genome-wide transcription targets for SET7/9

**Fig. 2:**
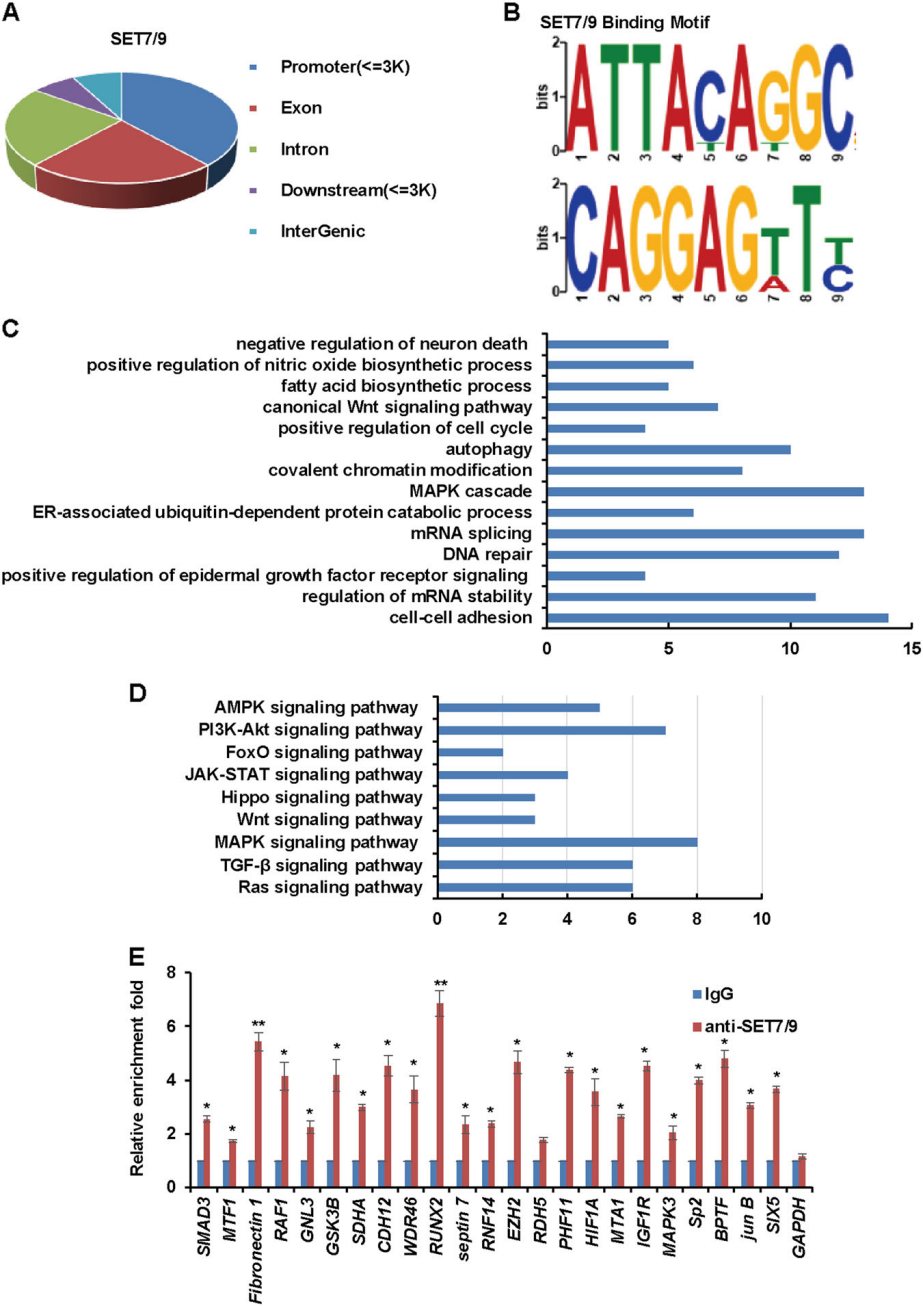
Identification of genome-wide transcription targets for SET7/9. **a** ChIP-seq analysis was performed in MCF-7 cells using a specific antibody against SET7/9, and the peaks’ distribution of SET7/9 was determined. **b** Discriminative regular expression motif elicitation was used to analyze the potential binding motif of SET7/9. **c** Cellular activities affected by SET7/9 according to Gene Ontology. **d** Kyoto Encyclopedia of Genes and Genomes (KEGG) pathway analysis was used to identify the pathways in which the potential target genes of SET7/9 were involved. **e** qChIP analysis was performed in MCF-7 cells using an anti-SET7/9 antibody to detect the binding of SET7/9 to the selected target genes with GAPDH as a negative control. Data are expressed as the fold change over the normal IgG group. Error bars represent the mean ± S.D. for three independent experiments. *P < 0.05; **P < 0.01.

As monomethylation at histone 3 lysine 4 by SET7/9 is generally associated with transcriptional activation^23,24^, we first focused our attention on the functional significance of SET7/9. The genome-wide transcriptional targets of SET7/9 were analyzed by chromatin immunoprecipitation-based deep sequencing (ChIP-seq) in MCF-7 cells. SET7/9-associated DNAs were amplified and then sequenced using the HiSeq 2000 platform with a *p* value cutoff of 10^–3^, and expression peaks were detected by model-based analysis for ChIP-Seq. We identified 28,271 SET7/9-specific binding sites, and the following peaks were identified in the genomic distribution: 38.34% promoters, 23.36% exons, 23.42% introns, 7.08% downstream (≤3 kb) regions, and 7.80% distal intergenic regions, as shown in Fig. 2a. Significantly, the binding motifs were identified (Fig. 2b). As the binding sites of SET7/9 in gene promoters were considered potential targets, the corresponding genes were classified according to Gene Ontology (GO) analysis, which implicated SET7/9 in the regulation of genes involved in cell-cell adhesion, DNA repair, the MAPK cascade, and the canonical Wnt signaling pathway (Fig. 2c). Furthermore, using the Kyoto Encyclopedia of Genes and Genomes Mapper, we identified several pathways that were significantly enriched, such as the MAPK, TGF-β, and PI3K-Akt pathways, which indicated that SET7/9 was critically involved in breast cancer cell growth, survival, migration, and invasion (Fig. 2d). Significantly, a quantitative ChIP (qChIP) assay was performed with antibodies against SET7/9 in MCF-7 cells targeting selected genes including RUNX2, EZH2, Fibronectin1, MTA1, and BPTF, which represented the classified pathways, remarkable enrichment on the promoters of these genes were observed, further confirmed the ChIP-seq result (Fig. 2e). As mentioned, RUNX2 is a member of the mammalian Runt-related transcription factor family and is recognized for its oncogenic properties in different types of human tumors including breast cancer^25–27^, therefore, we next focus our attention on the regulation of RUNX2 by SET7/9.

### SET7/9 promotes breast cancer carcinogenicity through activation of RUNX2

**Fig. 3:**
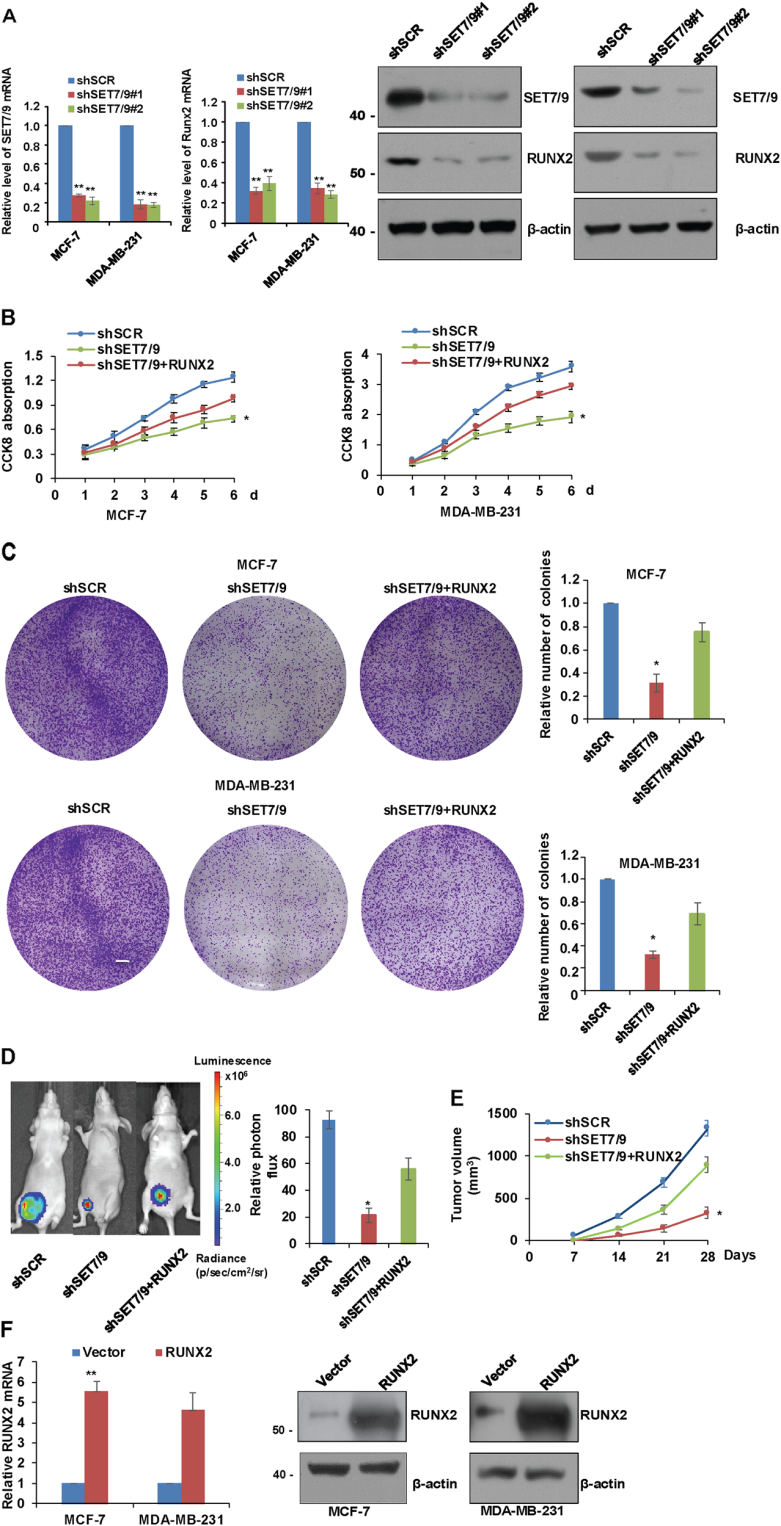
SET7/9 promotes breast cancer carcinogenicity through the activation of RUNX2. **a** The expression of SET7/9 and RUNX2 was detected by RT-qPCR and western blot in MCF-7 and MDA-MB-231 cell lines transfected with shSCR, shSET7/9#1, and shSET7/9#2. Data are presented as the mean ± S.D. *n* = 3. **P < 0.01. **b** CCK-8 analysis and **c** colony formation assays were used to detect cell proliferation in MCF-7 or MDA-MB-231 cells infected with shSCR, shSET7/9, or shSET7/9 plus RUNX2 at the indicated time points. *P < 0.05. **d** Xenograft tumors were quantified using bioluminescence imaging, and **e** volume was weekly when the tumors reached a size of ~1000 mm^3^ in all mice. Representative images are shown in the left panel, and the quantitative data are shown in the middle panel. The tumor volume was calculated at the indicated time points (right). *P < 0.05. **f** The expression of RUNX2 was detected by RT-qPCR and western blot in MCF-7 and MDA-MB-231 cell lines transfected with the vector alone or RUNX2. Data are presented as the mean ± S.D. *n* = 3. **P < 0.01.

As mentioned above, our genome-wide analysis indicated that SET7/9 targets several cellular signaling pathways that are involved in cell proliferation. Therefore, we further explore the function of SET7/9 in breast cancer cell proliferation and tumorigenesis. First, we knockdown the expression of SET7/9 in different breast cancer cell lines by lentivirus infection with two different SET7/9 short hairpin RNAs (shRNAs). The knockdown efficiency was monitored by RT-qPCR and western blot (Fig. 3a). Cells with SET7/9 knockdown exhibited a slower growth rate when compared with that of the control cells using CCK-8 assays (Fig. 3b). Consistent with this observation, colony formation assay demonstrated that depletion of SET7/9 was associated with a significantly reduced colony numbers of MCF-7 and MDA-MB-231 cells (Fig. 3c). To further establish the role of SET7/9 in promoting breast carcinogenesis, MDA-MB-231 cells engineered to stably express firefly luciferase were infected with SET7/9 shRNA lentiviruses or control shRNA lentiviruses. These cells were then orthotopically implanted onto the female nude mice for the measurements of tumor growth by using both quantitative bioluminescence imaging and tumor volume measurement weekly. The results showed that, knockdown of SET7/9 could significantly result in a dramatic reduction in tumor volume (Fig. 3d and Fig. 3e). In agreement with the hypothesis that RUNX2 might function as a target gene of SET7/9, the depletion of SET7/9 in MCF-7 cells and MDA-MB-231 cells led to decreased expression of RUNX2 (Fig. 3a). To clarify whether the knockdown of SET7/9 affected breast cancer cell growth in a RUNX2-dependent manner, we further ectopically overexpressed RUNX2 in the above cell lines. The transfection efficiency was confirmed by RT-qPCR and western blot analysis (Fig. 3f). Next, we monitored the proliferation capacity of breast cancer cells following the depletion of SET7/9 together with the overexpression of RUNX2. As shown in Fig. 3b–f, the overexpression of RUNX2 could significantly eliminate the blunted proliferation capacity of SET7/9-deficient cells.

### SET7/9 activates RUNX2 to enhance the migration and invasion potential of breast cancer cells

**Fig. 4:**
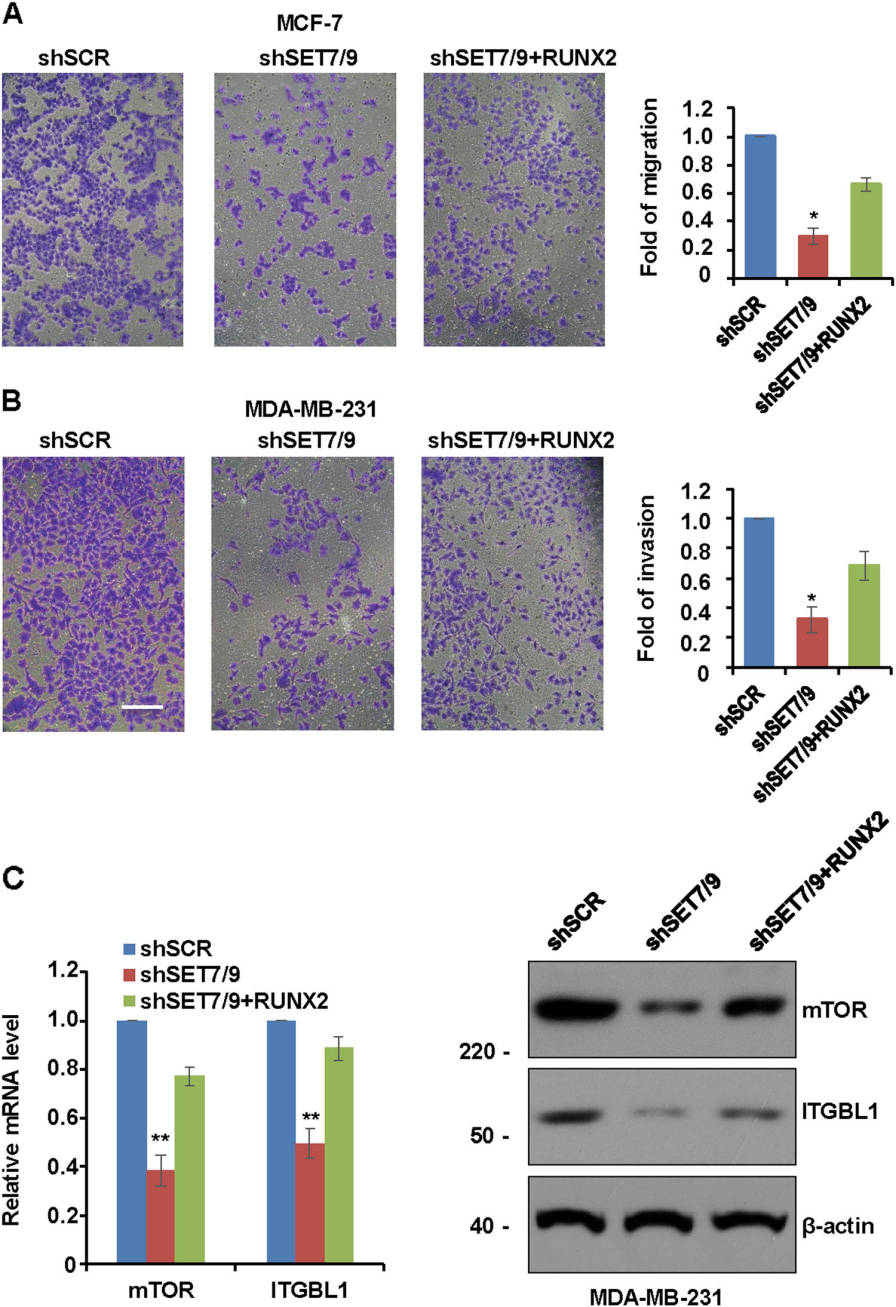
SET7/9 activates RUNX2 to increase the migration and invasion potential of breast cancer cells. **a** Transwell chamber filters were coated without Matrigel, and migration assays were performed with MCF-7 cells infected with shSCR, shSET7/9, or shSET7/9 plus RUNX2. **b** Transwell chamber filters were coated with Matrigel, and invasion assays were performed with MDA-MB-231 cells infected with shSCR, shSET7/9, or shSET7/9 plus RUNX2. Representative images are shown in the left panel, and quantitative data are shown in the right panel. Error bars represent the mean ± S.D. for three independent experiments. *P < 0.05. **c** The expression of mTOR and ITGBL1 was detected by RT-qPCR and western blot in MDA-MB-231 cell lines transfected with shSCR, shSET7/9, and shSET7/9 plus RUNX2. Data are presented as the mean ± S.D. *n* = 3. **P < 0.01.

To elucidate the function of SET7/9 and RUNX2 and their roles in breast cancer cell migration and invasion, a Transwell assay was performed in MCF-7 or MDA-MB-231 cells infected with SET7/9 shRNAs, and the results showed that the knockdown of SET7/9 robustly inhibited the migration activities of MCF-7 cells and the invasion capability of MDA-MB-231 cells. The overexpression of RUNX2 significantly reversed the inhibitory effect of shSET7/9 (Fig. 4a, b). As shown in Fig. 2d, several pathways including TGF-β, PI3K/Akt pathway might be involved in the promotion of breast cancer development by SET7/9. Interestingly, as reported by different groups^28,29^, Runx2 could activate PI3K/Akt signaling via mTOR regulation and activate the TGF-β signaling pathway through working as an upstream activator of ITGBL1. So, we next investigated whether the function of SET7/9 was through RUNX2 target genes such as mTOR and ITGBL1. As shown in Fig. 4c, the mRNA and protein level of mTOR and ITGBL1 was decreased in SET7/9-deficient breast cancer cells MDA-MB-231, whereas the overexpression of RUNX2 significantly eliminated the attenuation, which further indicated the effect of SET7/9 in breast cancer development was through RUNX2 and its target genes.

### TRIM21 is physically associated with SET7/9

**Fig. 5:**
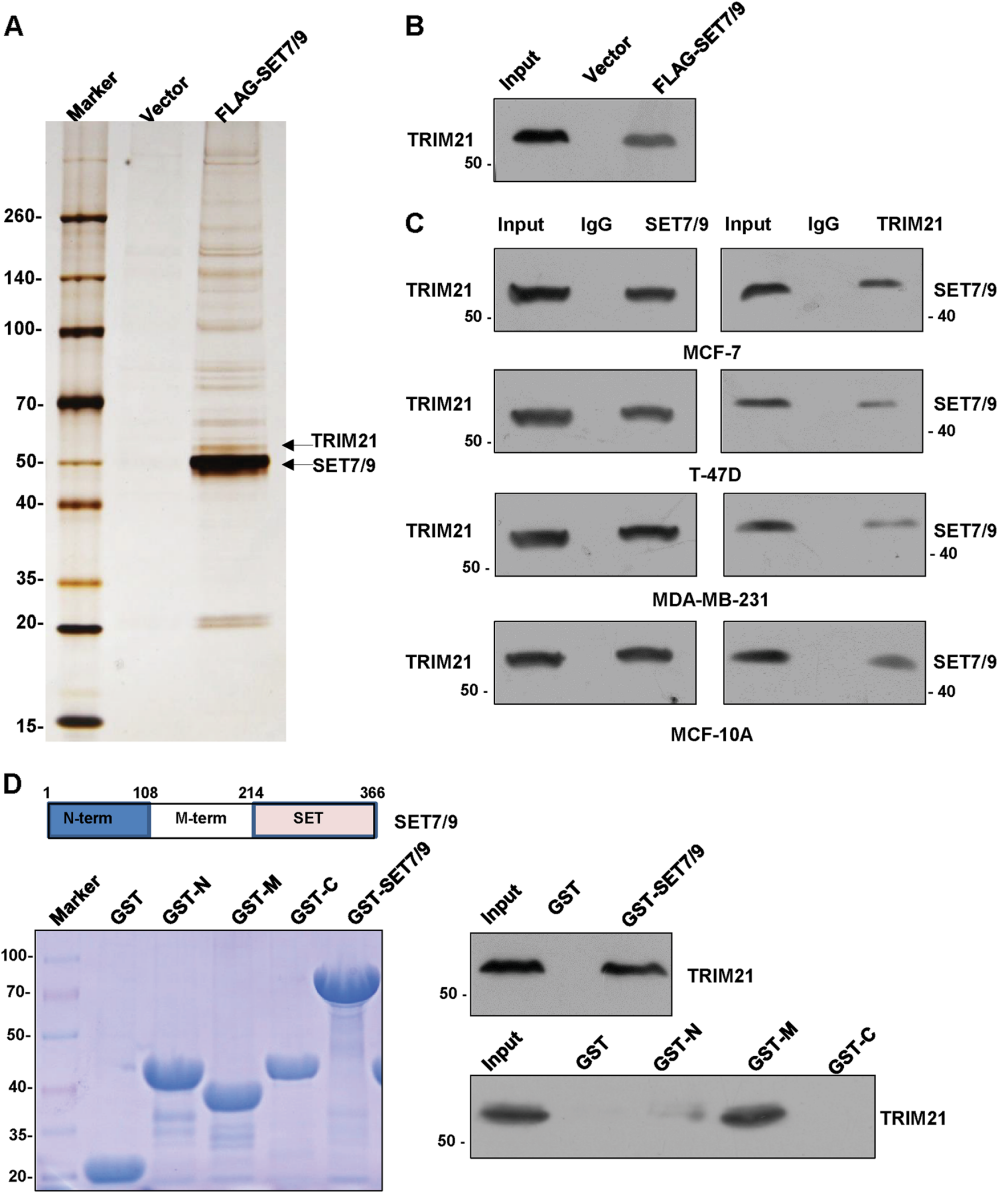
TRIM21 is physically associated with SET7/9. **a** Cellular extracts from MCF-7 cells stably expressing FLAG-SET7/9 or the vector alone were immunopurified with an anti-FLAG affinity column and eluted using FLAG peptides. The eluates were resolved by SDS–PAGE and analyzed by mass spectrometry. **b** The column-bound proteins were analyzed by western blot using antibodies against TRIM21. **c** Co-immunoprecipitation analysis was performed in MCF-7 cells, T-47D cells, MDA-MB-231 cells, and MCF-10A cells with antibodies against SET7/9. Immunocomplexes were then immunoblotted using antibodies against TRIM21, and the reciprocal procedure was also performed. **d** The GST pull-down assay was performed with GST-SET7/9 or its deletion mutants and in vitro-transcribed and translated full-length TRIM21.

To better understand the mechanistic role of SET7/9, affinity purification and mass spectrometry assays were performed to identify the proteins that were associated with SET7/9. First, MCF-7 cells stably expressing FLAG-SET7/9 were established, and anti-FLAG affinity gel was used to purify the MCF-7 cell extracts. Mass spectrometric analysis indicated that SET7/9 was co-purified with TRIM21 (Fig. 5a). The presence of TRIM21 was next confirmed by western blot (Fig. 5b). To further substantiate the interaction between SET7/9 and TRIM21, co-immunoprecipitation (co-IP)assays were carried out in MCF-7 cells, T-47D cells, MDA-MB-231 cells, and MCF-10A cells, respectively. IP with antibodies against SET7/9 followed by immunoblotting (IB) with TRIM21 antibodies revealed that TRIM21 was efficiently co-immunoprecipitated with SET7/9 (Fig. 5c, left panel). Reciprocally, IP with TRIM21 antibodies followed by IB with antibodies against SET7/9 also showed that SET7/9 was efficiently co-immunoprecipitated by TRIM21 (Fig. 5c, right panel). These results strongly indicated that SET7/9 was physically associated with TRIM21. In addition, a GST pull-down assay indicated that TRIM21 was capable of interacting with SET7/9 in vitro. To further determine which domain of SET7/9 mediated its interaction with TRIM21, we obtained three SET7/9 deletion constructs. Schematic representations of these constructs can be seen in Fig. 5d (upper panel). GST pull-down was further performed, and as shown, the middle terminal of SET7/9 was responsible for its interaction with TRIM21. Although the SET domain and the N domain of SET7/9 were not essential for the interaction of SET7/9 with TRIM21 (Fig. 5d).

### TRIM21 is an E3 ubiquitin ligase that specifically targets SET7/9 for degradation

**Fig. 6:**
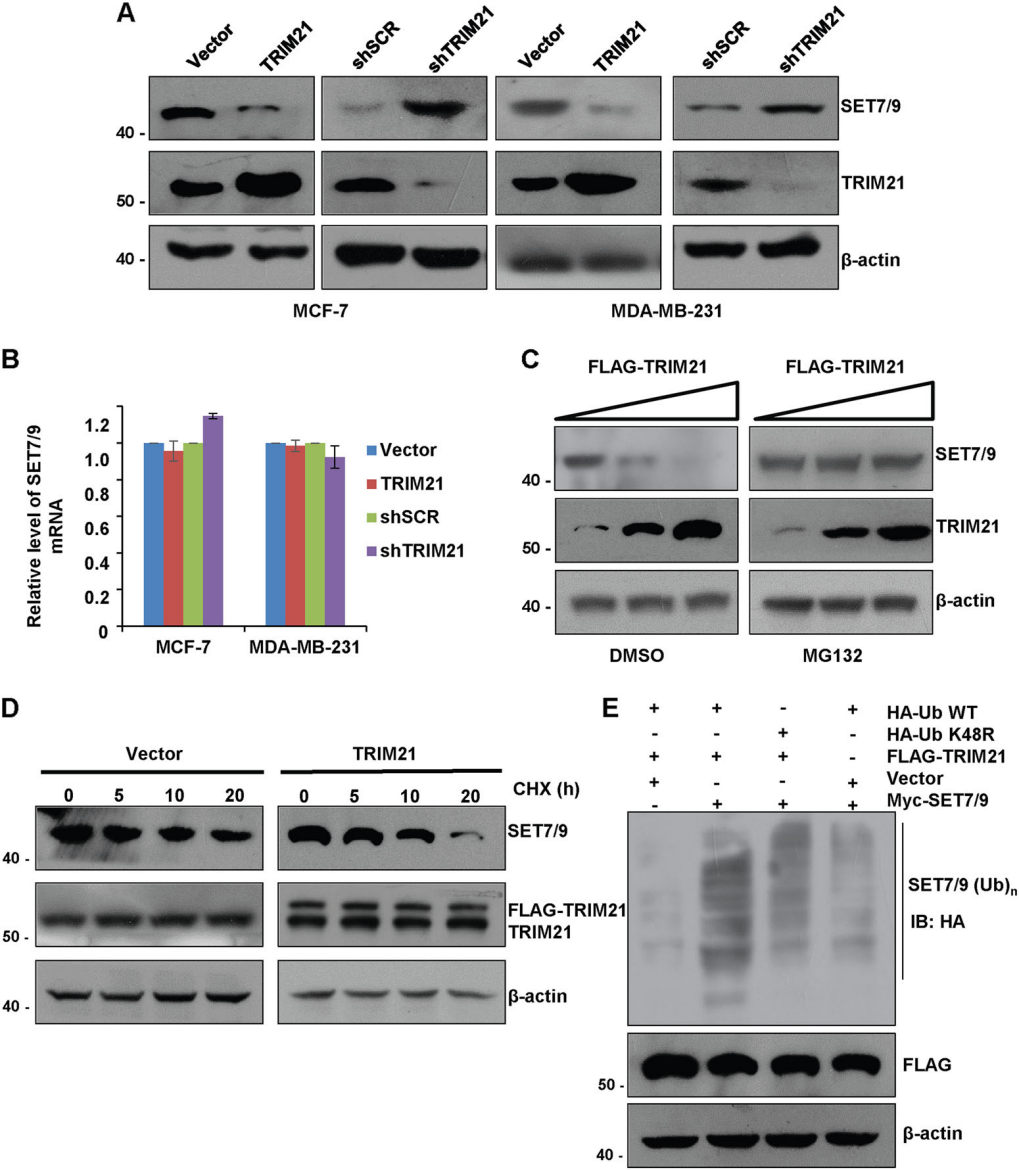
TRIM21 is an E3 ubiquitin ligase that specifically targets SET7/9 for degradation. **a** MCF-7 and MDA-MB-231 cells were transfected with the vector alone or FLAG-tagged TRIM21 or were treated with control shRNA or TRIM21 shRNA. Cellular extracts were collected, and western blot was performed with specific antibodies against the indicated proteins. **b** The cells from the experiments in **a** were analyzed for the expression of SET7/9 mRNA by RT-qPCR. Error bars represent the mean ± SD of triplicate experiments. **c** MCF-7 cells were transfected with the vector alone or FLAG-TRIM21 and then treated with DMSO or MG132 for 6 h before the cells were collected. The cell lysates were analyzed by western blot with antibodies against the indicated proteins. **d** MCF-7 cells were transfected with the vector alone or FLAG-TRIM21. Forty-eight hours after transfection, the cells were treated with 50 μg/ml cycloheximide (CHX) for the indicated times, and cellular proteins were extracted for western blot analysis. **e** MCF-7 cells were co-transfected with the indicated plasmids. Forty-eight hours after transfection, the cells were treated with MG132 for 12 h before cellular extracts were prepared for immunoprecipitation (IP) with anti-Myc followed by immunoblotting (IB) with anti-HA.

To investigate the hypothesis that TRIM21 is an E3 ubiquitin ligase for SET7/9, TRIM21 gain- and loss-of-function analyses were carried out in MCF-7 and MDA-MB-231 cells. Western blot analysis of the cellular lysates revealed that increased expression of TRIM21 was associated with a decreased level of the SET7/9 protein, whereas the depletion of TRIM21 led to an increase in the level of the SET7/9 protein (Fig. 6a). In particular, under TRIM21 overexpression, a decreased SET7/9 protein level did not result from SET7/9 mRNA downregulation (Fig. 6b). Furthermore, the TRIM21-associated destabilization of the SET7/9 protein could be effectively blocked by MG132, a proteasome inhibitor (Fig. 6c), suggesting that this process was probably mediated by the ubiquitin–proteasome pathway. Cycloheximide (CHX) chase experiments were carried out in MCF-7 cells transfected with TRIM21 or the vector alone. Western blot analysis revealed that the overexpression of TRIM21 was associated with a decrease in the half-life of SET7/9 (Fig. 6d). To further strengthen the proteasome-dependent mechanism of TRIM21-mediated SET7/9 degradation, we next determined whether the overexpression of TRIM21 could promote SET7/9 ubiquitination. To this end, MCF-7 cells were co-transfected with FLAG-TRIM21 together with Myc-SET7/9 and HA-tagged wild-type ubiquitin or HA-tagged ubiquitin mutants (UbK48R). The IP of the cellular lysates with a specific Myc antibody followed by IB with a specific HA antibody indicated that TRIM21 could promote SET7/9 polyubiquitination (Fig. 6e). Together, these results support the notion that TRIM21 specifically targets SET7/9 for degradation through a ubiquitination-mediated process.

### TRIM21 inhibits breast cancer development via SET7/9 and is associated with a good prognosis

**Fig. 7:**
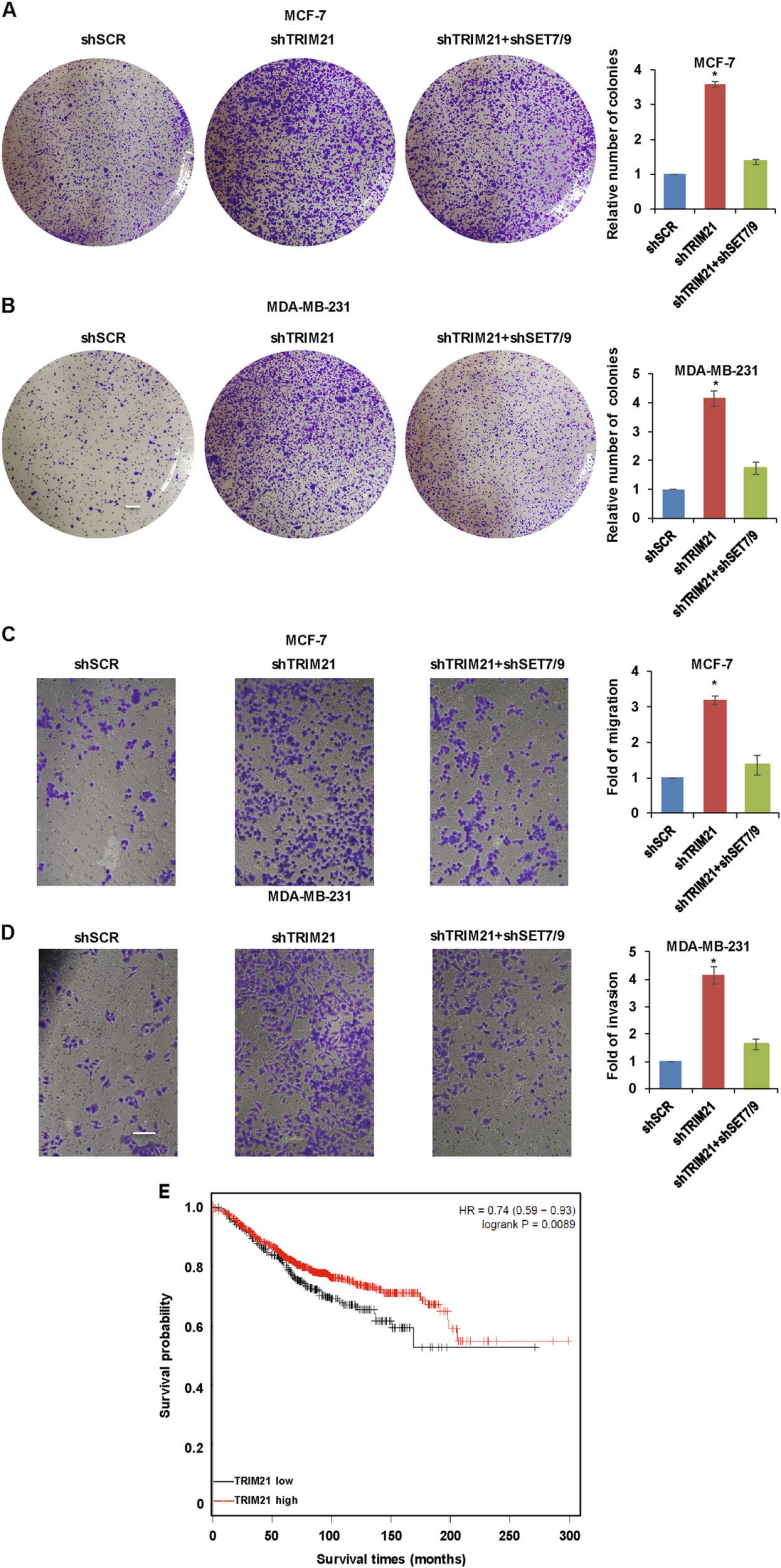
TRIM21 inhibits breast cancer development through SET7/9 and is associated with a good prognosis. **a** CCK-8 analysis and **b** colony formation assay were used to detect cell proliferation in MCF-7 or MDA-MB-231 cells transfected with the vector alone, the shTRIM21 construct, or the TRIM21 and SET7/9 double knockdown construct. **c** Migration assays were performed in MCF-7 cells, and invasion assays were performed in MDA-MB-231 cells **d** transfected with the vector alone, the shTRIM21 construct, or the TRIM21 and SET7/9 double knockdown construct. **e** A Kaplan–Meier plot was used to show the association between TRIM21 mRNA expression and breast cancer patients’ overall survival times.

**Fig. 8:**
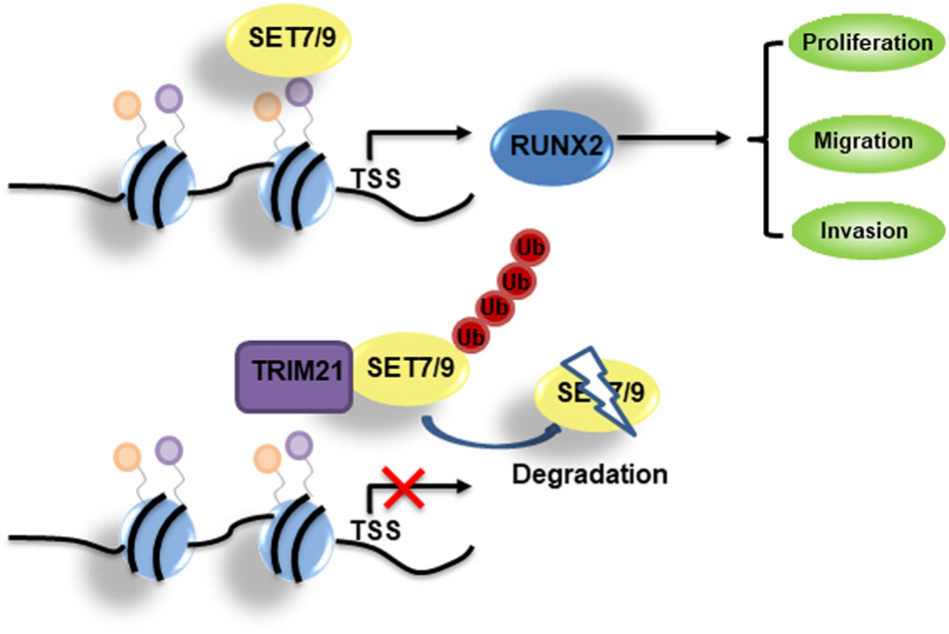
Proposed model for the role of SET7/9 in breast cancer cells. The mechanism of SET7/9-mediated breast cancer cell proliferation, migration, and invasion is mediated by the noted activation of RUNX2. In addition, SET7/9 is specifically recognized by TRIM21 and degraded by the ubiquitination system.

To monitor the biological significance of TRIM21 in mediating SET7/9 degradation, CCK-8 and colony formation assays were performed in MCF-7 cells or MDA-MB-231 cells. TRIM21 depletion was associated with increased effects on the proliferation of breast cancer cells, which were partially attenuated by the simultaneous knockdown of SET7/9 (Fig. 7a, b). Similarly, MCF-7 and MDA-MB-231 cells with TRIM21 and SET7/9 double knockdown showed weakened migration and invasion capabilities compared with those subjected to TRIM21 knockdown alone (Fig. 7c, d). Together, these results demonstrated that TRIM21 regulated breast cancer progression via SET7/9. Accordingly, the online survival analysis database Kaplan–Meier plotter demonstrated the prognostic value of TRIM21 mRNA in breast cancer according to the mRNA gene chip data. As shown in Fig. 7e, elevated TRIM21 mRNA levels were associated with better overall survival times (hazard ratio, 0.74; *P* = 0.0089). In summary, we explored the mechanism of SET7/9-mediated breast cancer cell proliferation, migration, and invasion through the activation of RUNX2. SET7/9 was shown to be specifically recognized by TRIM21 and degraded by the ubiquitination system (Fig. 8).

## Discussion

The stimulation of transcriptional activation involves numerous diverse classes of proteins known as transcription coactivators^30,31^. SET7/9 catalyzes the monomethylation of histone H3 lysine 4 (H3K4me1) and non-histone proteins such as serum response factor in embryonic stem cells to regulate gene expression as coactivators^32^. In breast cancer, as reported by Subramanian K. et al.^33^, estrogen receptor α (ER) is a ligand-dependent transcription factor that can be directly methylated by SET7/9. SET7/9-mediated ER methylation is necessary for ER to activate downstream target genes. Vascular endothelial growth factor (VEGF) is one of the most important regulators of tumor angiogenesis in breast cancer^34^. As Zhang Y. et al.^35^ reported that SET7/9, which is recruited by the transcription factor GATA1, can promote VEGF transcription and breast tumor angiogenesis. This evidence indicates that SET7/9 in breast cancer might function as an oncogene through the methylation of histones to activate target gene expression or the methylation of non-histone proteins directly. In our study, we revealed that through the activation of RUNX2, SET7/9 could promote breast cancer progression in vitro and in vivo. Our data also showed that SET7/9 was upregulated in breast cancer tissues and cell lines. An increase in SET7/9 is closely associated with poor prognosis.

RUNX2 is necessary during embryogenesis^36^ and is involved in many pathways, including Wnt and TGFβ signaling pathways, which are important in the development of different types of cancer, including breast cancer^37,38^. It is now believed that RUNX2 has a role in cell cycle promotion by interacting directly with the p53 tumor suppressor or negatively regulating the cell cycle inhibitor pRB^39,40^. The overexpression of RUNX2 in MCF-7 cells induced epithelial-to-mesenchymal transition (EMT) and promoted breast cancer cell invasion in vitro^41^. RUNX2 influences a number of genes involved in cancer progression, such as BSP, MMPs, or VEGF^26^. During osteogenic differentiation, monoubiquitination by WWP2 leads to RUNX2 transactivation^42^. In our study, we reveal a new model to illuminate the contribution of the activation of RUNX2 expression by SET7/9 in breast cancer to carcinogenicity. Although in colony formation assay, MTA1 overexpression could partially reduce the colony formation capacity of SET7/9-deficient cells (Supplementary Figure 1), more evidences are needed to confirm whether more potential target genes including MTA1 are involved in the breast cancer progress. In this study, we mainly focus on the target of RUNX2 by SET7/9 contributes to breast cancer progression.

Emerging clinical evidence has shown that ubiquitin-mediated degradation functions as an important regulator of carcinogenesis^43,44^. Although TRIM21 was first identified as a regulator of innate immune signaling, more and more evidences suggested a possible tumor suppressive role of TRIM21 in breast cancer. As Zhou W. et al.^45^ reported, decreased expression of TRIM21 indicates unfavorable outcome and promotes cell growth. According to Jin Y.^46^, TRIM21 could interact with Snail, a master regulator of EMT, and lead to increased ubiquitination degradation of Snail in breast cancer cells MCF-7 and T-47D, these evidences are consistent with us that TRIM21 is a potential tumor suppressor in breast cancer. Strikingly, we identified that SET7/9 was another substrate of TRIM21, by mediating the proteosomal degradation of SET7/9, TRIM21 could suppress the proliferation, migration, and invasion of breast cancer cells. The interpretation of TRIM21/SET7/9/RUNX2 axis leads to a deeper comprehend about the molecular properties of breast cancer malignancy, and might be an attractive target for its diagnosis and therapy.

In summary, we identified a critical role of SET7/9 as important oncogene in the development of breast cancer. Abnormally elevated SET7/9 expression could enhance breast cancer proliferation migration and invasion via activation of RUNX2 from transcription level. Moreover, TRIM21 worked a negative regulator to suppress SET7/9 expression through proteasome-dependent mechanism. The TRIM21-SET7/9-RUNX2 axis might potentially be a novel target for the treatment of breast cancer.

## Materials and methods

### Antibodies and reagents

Antibodies used: αRUNX2 (12556) was obtained from Cell Signaling Technology; αSET7/9 (ab14820), αmTOR (ab2732), αITGBL1 (ab251678), and β-actin (ab227387) was purchased from Abcam; αTRIM21 was obtained from Santa Cruz Biotechnology. Protein A/G Sepharose CL-4B beads were purchased from Amersham Biosciences, and a protease inhibitor cocktail was obtained from Roche Applied Science.

### Cell culture

The breast cancer cell lines MCF-7, MDA-MB-231, MDA-MB-468, T-47D, UACC-812, and MCF-10A were obtained from the American Type Culture Collection (ATCC, Manassas, VA, USA). MCF-7, T-47D, MDA-MB-468 cells were maintained in Dulbecco's Modified Eagle Medium (DMEM; GIBCO, NY, USA) supplemented with 10% fetal bovine serum (FBS; HyClone). MCF-10A cells were cultured in MEBM BulletKit (Lonza, Basel, Switzerland). The two cell lines were cultured in a humidified incubator equilibrated at 37 °C in 5% CO_2_. MDA-MB-231 and UACC-812 cells were cultured in L-15 (GIBCO) with 10% FBS without CO_2_, supplemented with antibiotics (100 U/ml penicillin and 100 mg/ml streptomycin; Sigma-Aldrich, USA). The cell lines in the experiments were validated by STR DNA analysis and were negative for mycoplasma.

### Lentiviral production and infection

Recombinant lentiviruses expressing shSET7/9, shTRIM21, shSCR, FLAG-SET7/9, FLAG-TRIM21, FLAG-RUNX2, or FLAG-Vector were obtained from Shanghai GenePharma and used according to the manufacturer’s instructions. The concentrated lentiviruses were used to infect cells with 8 μg/ml polybrene in a 60-mm dish (5 × 10^5^ cells per well). The cells were selected with 2 μg/ml puromycin (Merck) and subjected to sorting.

### Silver staining and mass spectrometry

MCF-7 cells stably expressing FLAG-SET7/9 or FLAG-Vector were produced by the infection of the cells with the FLAG-tagged SET7/9 or FLAG-Vector lentivirus. Anti-FLAG M2 affinity gel was purchased from Sigma, and anti-FLAG immune affinity columns were prepared according to the manufacturer’s suggestions. The precipitated FLAG protein complex was eluted from the column with the FLAG peptide (0.2 mg/ml, Sigma-Aldrich). The elution fractions were collected and resolved on sodium dodecyl sulfate–polyacrylamide gel electrophoresis (SDS–PAGE) gels, silver staining was carried out to detect the bands using the Pierce Silver Stain Kit (Thermo Fisher Scientific). The bands were excised and subjected to LC-MS/MS sequencing and data analysis.

### Co-IP analysis

Cells were lysed by incubating the cells with lysis buffer (50 mm Tris-HCl, pH 8.0, 0.5% NP40, 150 mm NaCl, 1 mm EDTA, 0.5% sodium deoxycholate, and a protease inhibitor cocktail) for 40 min on a rotator at 4 °C. Following centrifugation at 13,000 rpm for 15 min at 4 °C, equal quantities of the supernatants from each group were incubated with 3 μg of primary antibodies or normal rabbit/mouse IgG as a negative control on the rotator overnight at 4 °C. Then, 60 μl of 50% protein A/G Sepharose CL-4B beads was added to the immune complexes, and incubation was continued for an additional 2 h at 4 °C. After washing five times using lysis buffer, the immune complexes were eluted using 2× SDS–PAGE loading buffer and subjected to SDS–PAGE, followed by western blot analysis.

### Western blot analysis

The materials obtained from IP or equal amounts of protein (30 μg) from the cell lysates were resolved using 8–15% SDS–PAGE gels and transferred to polyvinylidene difluoride membranes. As the formula weight of β-actin and SET7/9 is close to each other, the details of the western blot analysis were performed as follows: first, the membrane was incubated with SET7/9 antibody (ab14820, abcam), the source of which is from mouse; then incubated with the corresponding goat anti-mouse IgG H&L secondary antibodies and visualized. After washing with striping buffer and TBST for five times, the membrane was blocked with 5% non-fat milk again, and then incubated with β-actin antibody, the source of which is from rabbit. The membrane was then incubated with the corresponding goat anti-rabbit IgG H&L secondary antibodies and visualized using western blot luminol reagent (Santa Cruz Biotechnology) according to the manufacturer’s recommendation.

### GST pull-down assay

GST pull-down assays were performed as described earlier^47^. The in vitro transcription and translation experiments were performed with a rabbit reticulocyte lysate kit (Promega).

### RNA isolation and real-time reverse transcription polymerase chain reaction

Total RNA was isolated using TRIzol solution (Invitrogen Life Technologies, Carlsbad, CA, USA) according to the manufacturer’s instructions. One microgram of total RNA was used to synthesize first-strand cDNA with the reverse transcription system (Roche). Quantitative real-time PCR was carried out with Power SYBR Green PCR Master Mix (Roche) on an ABI 7500 Real-Time detection system (Applied Biosystems, USA), and glyceraldehyde 3-phosphate dehydrogenase (GAPDH) was used as a control. The amplification protocol was as follows: initial denaturation at 95 °C for 5 min, followed by 40 cycles of denaturation at 95 °C for 15 s and annealing and extension at 60 °C for 30 s. The experiments were repeated at least three times independently.

### ChIP-seq and qChIP

Approximately 5 × 10^7^ MCF-7 cells in each group were used for the ChIP-seq assay according to a previous protocol^47^. The chromatin DNA was precipitated with either normal rabbit IgG (control) or SET7/9 antibodies. The Qiagen PCR purification kit was used to purify the DNA. In-depth whole-genome DNA sequencing was performed by the CapitalBio Corporation, Beijing. Analyses at the Gene Ontology (GO) (http://www.geneontology.org) and Kyoto Encyclopedia of Genes and Genomes (KEGG) (http://www.genome.jp/kegg/) databases were performed. Significance was considered to be indicated by a *p* value cutoff of 0.05. The investigator was blinded to the group allocation during the experiment, ChIP-seq data are deposited at the Gene Expression Omnibus database. qChIP detection was performed using TransStart Top Green qPCR Supermix (TransGen Biotech, Beijing, China).

### CCK-8 assay

After infection with the relevant lentivirus, the cultured MCF-7 and MDA-MB-231 cells were trypsinised and suspended, and 3000 cells were seeded into each well of a 96-well plate. Every 24 h, 10 μl of CCK-8 reagent was added to each well, followed by incubation for another 2 h. Then, the absorbance was measured in a microplate reader (ELx800; Biotek, Winooski, VT, USA). The proliferation curves were drawn using the OD values. Each experiment was performed in triplicate.

### Colony formation assay

MCF-7 cells and MDA-MB-231 cells were stably infected with the appropriate lentiviral expression vector. The cells were cultured in the media for 2 weeks with 2 μg/ml puromycin and were then stained with crystal violet.

### Cell migration and invasion assays

Transwell chamber filters coated with or without Matrigel (BD Biosciences) were prepared. The migration assay procedure was similar to that of the invasion experiment, except for the Matrigel coating. After infection with the relative lentivirus, 2.5 × 10^5^ MCF-7 cells were seeded into the upper Transwell chambers containing 0.5 ml DMEM without FBS, whereas the lower chambers contained 1 ml DMEM with 10% FBS. For MDA-MB-231 cells, the medium was L-15 with or without FBS. After incubation for 24 h, the cells in the top well were removed using cotton swabs, while cells that migrated or invaded through to the other side of the chamber were stained and counted under a microscope. The experiments were performed in triplicate.

### Bioluminescent imaging

The MDA-MB-231-Luc-D3H2LN cell line was purchased from Xenogen Corporation. After infection with the appropriate recombinant lentivirus, these cells were inoculated into the left abdominal mammary fat pad (3 × 10^6^ cells) of 8-week-old female nude mice (Charles River, Beijing, China), each group including eight mice, and the animals were allocated to experimental groups using method of randomization. The details of the bioluminescence imaging procedure were as described earlier^48^. The animal procedures were approved by the Peking University Health Science Center Institutional Animal Care and Use Committee.

### Statistical analysis

All statistical analyses were performed using SPSS 17.0 software (SPSS, USA). Adequate sample size was determined according to the previous studies that performed analogous experiments. The results was normally distributed and presented as the mean ± S.D. and analyzed using Student’s *t* test for two groups or one-way analysis of variance for more than two groups (followed by the post hoc Tukey–Kramer test) unless otherwise noted. For animal studies, no blinding was used. Kaplan–Meier curves were used to examine the association between the expression of SET7/9 and survival times. *P* < 0.05 was considered statistically significant. The experiments were repeated at least three times.

## Supplementary information

Supplementary figure legends

Supplementary figures

## Availability of data and materials

The analyzed data sets are available from the corresponding author, on reasonable request.
