# Supplementary material for: SET7/9 promotes multiple malignant processes in breast cancer development via RUNX2 activation and is negatively regulated by TRIM21
Source: Cell Death Dis. 2020 Feb 26;11(2):151. doi: 10.1038/s41419-020-2350-2 (PMC7044199; doi:10.1038/s41419-020-2350-2)
Supplement: Supplementary file 1 — Supplementary figure legends [file 41419_2020_2350_MOESM1_ESM.docx]

**Supplementary Figure legends**

**Supplementary Figure 1.** MTA1 overexpression reverses the effect of SET7/9 in breast cancer cells *in vitro*. Colony formation assays were used to detect cell proliferation in MCF-7 or MDA-MB-231 cells infected with shSCR, shSET7/9, or shSET7/9 plus MTA1. *P < 0.05.
