# Supplementary figures and images for: SET7/9 promotes multiple malignant processes in breast cancer development via RUNX2 activation and is negatively regulated by TRIM21
Source: Cell Death Dis. 2020 Feb 26;11(2):151. doi: 10.1038/s41419-020-2350-2 (PMC7044199; doi:10.1038/s41419-020-2350-2)

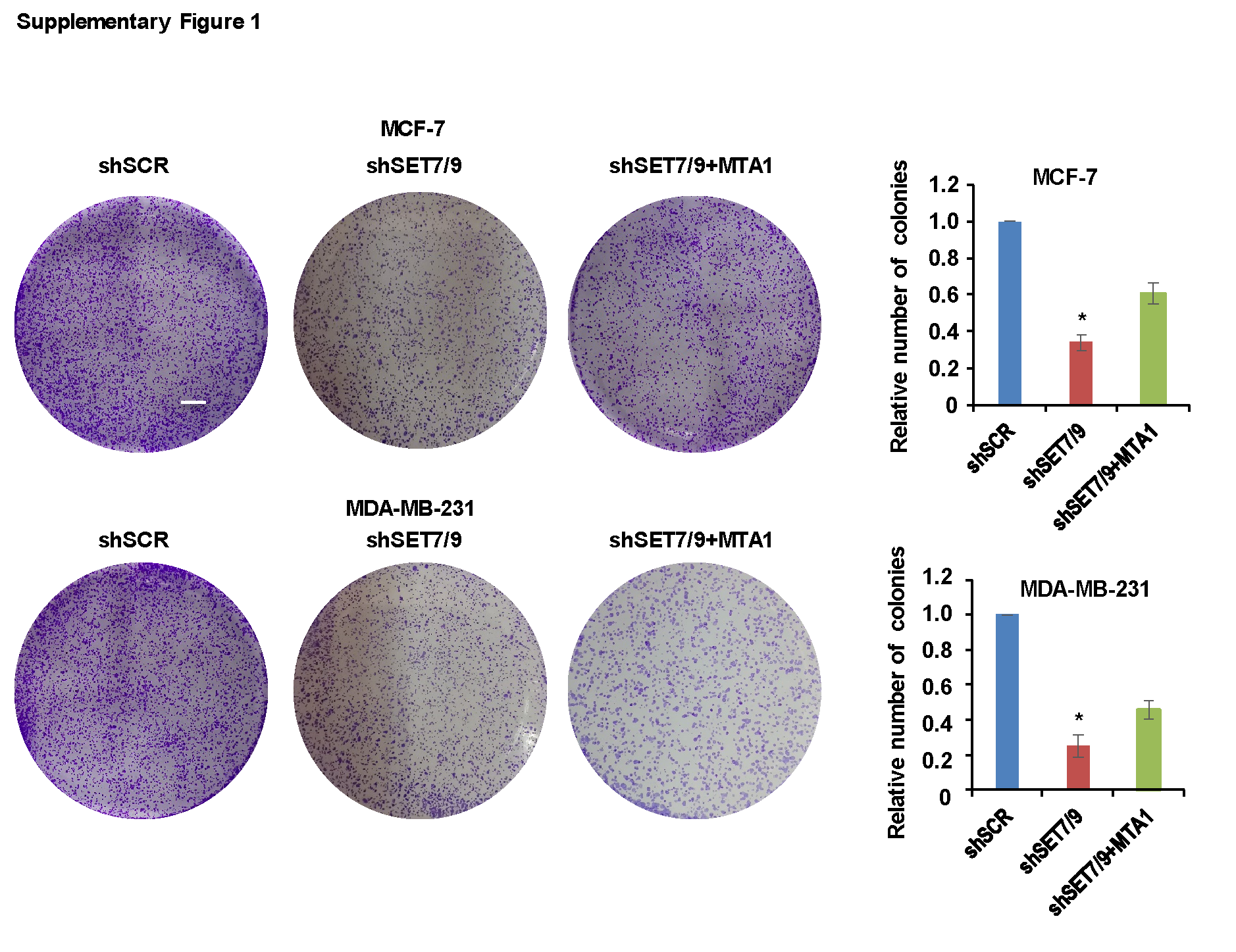

Supplement: Supplementary file 2 — Supplementary figures [file 41419_2020_2350_MOESM2_ESM.tif]
